# The Back-care Behavior Assessment Questionnaire (BABAQ) for schoolchildren: development and psychometric evaluation

**DOI:** 10.1186/s12889-020-09318-9

**Published:** 2020-08-26

**Authors:** Zahra Akbari-Chehrehbargh, Sedigheh Sadat Tavafian, Ali Montazeri

**Affiliations:** 1 Department of Health Education, Faculty of Medical Sciences, Tarbiat Modares University, Tehran, Iran; 2 Health Metrics Research Center, Iranian Institutes for Health Sciences Research, ACECR, Tehran, Iran; 3 Faculty of Humanity Sciences, University of Science &Culture, ACECR, Tehran, Iran

**Keywords:** Social cognitive theory (SCT), Spine-related behavior, Psychometric properties, Iran, Schoolchildren

## Abstract

**Background:**

Back pain is an important public health problem and the leading cause of adult disability worldwide and is rising among schoolchildren populations. Despite numerous studies reporting on back care interventions in pediatric population; there is currently no existing theory-based instrument to assess impact and outcome of these programs. This paper reports on development and psychometric testing of a theory based back-care behavior instrument for use among elementary schoolchildren.

**Methods:**

This was a three-phases study that included the following steps: a) a literature research to review existing instruments that assess healthy spine-related behavior in elementary schoolchildren; b) development of a new instrument namely the Back-care Behavior Assessment Questionnaire (BABAQ) based on the Social Cognitive Theory and existing instruments, and c) conducting a cross sectional study to test psychometric properties of the BABAQ by estimating the content validity ratio (CVR), the content validity index (CVI), performing confirmatory factor analysis (CFA), reliability analysis, and convergent validity as estimated by the Average Variance Extracted (AVE).

**Results:**

First, a questionnaire (the BABAQ) was developed. It contained of 49 items tapping into 5 pre-defined constructs (skills, knowledge, self-efficacy, expectation beliefs, and behavior). Then, 610 fifth-grade female schoolchildren were entered into a cross sectional study and they completed the BABAQ. The CVR and the CVI of the questionnaire was found to be ≥0.54 and > 0.7, respectively. The CFA confirmed the five constructs and showed good fit for the data. The intraclass correlation (ICC) and the Cronbach’s alpha coefficients for the BABAQ were 0.84 (*P* < 0.001) and 0.93, respectively. The convergent validity as measured by the AVE also showed satisfactory results.

**Conclusion:**

The findings suggest that the Back-care Behavior Assessment Questionnaire (BABAQ) is a valid instrument for measuring healthy spine-related behaviors among schoolchildren.

## Background

Musculoskeletal disorders (MSDs), including back pain, are among the most important problems causing excessive absenteeism in the workplace, imposing high economic costs on health care systems, and suffering nearly 540 million people [1–5]. As described by the World Health Organization (WHO), back pain comprises low back and neck pain (mild, moderate, severe, and most severe). An individual who develops back, leg, and arm pain might thus experience difficulty dressing, sitting, standing, walking, turning one’s head, holding arms up, as well as lifting things. They might also sleep poorly, have headaches, feel tired and worried, and lose some enjoyments of life [6]. Although the burden of back pain among adults has been thus far well documented, this subject matter in children is underreported. According to the WHO statistics in 2015, back pain ranked 9th place in years living with disability in 10-to-14-year-olds and 4th in children and young adolescents aged 15–19 years, even much higher than non-communicable diseases such as cancer and anxiety disorders [7]. It is of note that the lifetime prevalence rate of low back pain (LBP) in children varies from 13 to 51% [8] and increases with age wherein a sharp rise is evident. As transition occurs from childhood to adolescence, the boundary is approximately at the age of 10–13 years. In addition, previous studies have reported higher prevalence rates among adolescent girls than boys (38.9% vs. 35.0%) [3, 9]. As such, implementation of educational interventions for back-care among children and young adolescents are increasingly becoming popular. Therefore, it is argued that measuring healthy spine-related behaviors during daily life activities among children, as a key outcome in evaluation of educational interventions for back-care, is of prime importance [10].

Up until now, a number of questionnaires have been developed for such purposes. For instance, Spence et al. [11] and Sheldon [12] introduced written and practical tests to assess pupils’ knowledge and performance with regard to correct lifting techniques among 3th, 5th, 6th, and 8th-grade public-school children. As well, Monfort et al. [1] developed and evaluated the psychometric properties of a health questionnaire on back-care knowledge in daily life physical activities (known as HEBACAKNOW), consisting of 24 items examining levels of back-care knowledge among adolescents. Similarly, Noll et al. [2] designed the Back Pain and Body Posture Evaluation Instrument (BackPEI) for schoolchildren, relevant to the evaluation of back pain and its associated behavior risk factors. In addition, Cardon et al. [13–15] utilized a battery of questionnaires consisting of different constructs including general and specific back-care knowledge, fear-avoidance beliefs, self-efficacy, attitudes, self-reported behaviors, practical tests, social support, program commitment, and perceived behaviors for children, parents, and teachers.

Despite the effectiveness of such questionnaires in advancing knowledge on the subject matter, none has been theory-based. In addition, some discrepancies have been also found for constructs and psychometric properties of the questionnaires introduced. In fact, assessment of back-care behavior has been scarcely investigated from the theoretical point of view and most of the previous studies have not reflected on construct validity, especially, exploratory or confirmatory factor analyses (namely, EFA and CFA).

To this end we believe that despite numerous studies reporting on back care intervention in pediatric populations [8, 11, 13, 14], there is currently no existing a theory-based measure to assess impact and outcome of these programs. Thus, this study aimed to develop a theory based back-care behavior assessment questionnaire for pupil populations attending elementary schools. The specific objectives were to evaluate: content, face, and structural validity as well as reliability of its subsections.

## Methods

### Theoretical framework

The conceptual framework for this study and development of an instrument was based on the Social Cognitive Theory (SCT). It has been shown that this theory has a good power to predict behavior changes especially in pupils [16]. According to the SCT, three main psychological determinants of any behavior changes are: self-efficacy (SE); behavioral capability (skills and knowledge to perform a given behavior); and outcome expectation beliefs (behavioral beliefs) [17, 18]. The proposed cognitive factors of behavior are important set of modifiable factors that are assumed to combine in different ways to determine health related behavior and distinguish between those performing and not performing behaviors [17, 18]. Therefore, we thought an instrument that intends to measure back care behavior among elementary schoolchildren should address the constructs that proposed by this theory in order to achieve the desired behavior change of back care during daily activity.

### Design and procedure

This study comprised of three parts: a broad literature searches in order to review existing questionnaires for assessing of healthy spine-related behavior in elementary schoolchildren; compiling items to fulfill pre-defined constructs based on the social cognitive theory; and conducting a cross sectional study in order to validate the questionnaire among 5th-grade students attending elementary schools in Tehran, Iran.

### Preliminary questionnaire

The early version of the Back-care Behavior Assessment Questionnaire (BABAQ) was developed based on the content of other existing questionnaires (Table 1). The draft instrument yielded 55 items in five predefined constructs as follows:

1. A checklist for practical assessment of skills for back care principles. The checklist consisted of seven tasks and 24 items. Each item is rated on a 3-point scale ranging from 0 (not fulfilling the criteria) to 2 (correct completion of the task) giving score ranging from 0 to 48 points where higher scores indicate better fulfillment of tasks [14, 19].
2. Back care knowledge containing 13 multiple-choice questions. Scores on this construct range from 0 to 13 where the higher scores indicate better knowledge [12–14, 19].
3. Self-efficacy subscale containing 4 items. Each item is rated on a four-point scale (from difficult to easy) giving score ranging from 4 to 16 where the higher scores indicate higher self-efficacy [10, 13].
4. Expectation beliefs containing 6 items. Each item is rated on a five-point scale (strongly disagree to strongly agree) giving score ranging from 6 to 30 where higher score indicate stronger beliefs [10, 13].
5. Back care behavior containing 8 items regarding daily activity. Response categories ranged from never (1) to ever (5) giving a score ranging from 8 to 40 where higher scores indicate better preventive behavior [10, 13].

**Table 1:** Description of the constructs and the related citations that served as a basis for item generation for the Back-care Behavior Assessment Questionnaire (BABAQ)

Construct	Items	References
**Skills (check list)-Score sheet for the practical assessment**		
	Sitting at a table	Spence et al. (1984), Sheldon et al. (1994), Cardon et al. (2000, 2001, 2002, 2007), Heiser et al. (2014) and Santos et al. (2017)
	Pick up the crate	
	Carry the crate	
	Set the crate down on the table	
	Pick up a pencil	
	Move the crate	
	Book bag use	
**Knowledge**		
	General & specific back care knowledge	Spence et al. (1984), Sheldon et al. (1994), Cardon et al. (2000, 2001, 2002, 2007), Dolphens et al. (2011), Park et al. (2011), Heiser et al. (2014), Monfort-Pan˜ego et al. (2016) and Santos et al. (2017)
**Self-Efficacy**		
	How easy or difficult:	Cardon et al. (2002), Park et al. (2011) and Dolphens et al. (2011)
	Daily participation in physical activity	
	Attaining a natural curve of the spine	
	Minimal loading of the book bag	
	Pay attention to ergonomic postures	
**Expectations beliefs**		
	Sitting is ‘dangerous’ when having a backache	Cardon et al. (2002) and Dolphens et al. (2011)
	Swimming is ‘dangerous’ when having a backache	
	Running is ‘dangerous’ when having a backache	
	Participation in physical education is ‘dangerous’ when having a backache	
	Cycling is ‘dangerous’ when having a backache	
	Lifting heavy objects is ‘dangerous’ when having a backache	
**Self-reported behavior**		
	Checking weight of book bag	Cardon et al. (2002), Dolphens et al. (2011) and Noll et al. (2013)
	Carrying the bag with 2 straps	
	Elevator use versus taking the stairs	
	Knee position when putting on shoes	
	Doing exercises every day	
	Knee position when lifting	
	Distance to body when load carrying	
	No twisting while moving heavy object	
	Posture in relation to sleeping, sitting in a chair to write, sitting in a chair to talk, using a computer and lifting an object from the ground (BackPEI)	

Then, content and face validity of the preliminary version of the questionnaire was assessed. To determine the content validity, a panel of 13 specialists in health education and health promotion, epidemiology and physiotherapy reviewed the questionnaire in order to estimate the content validity ratio (CVR) and the content validity index (CVI). They rated items based on three evaluation options: unnecessary, useful but unnecessary, and necessary. The CVR was then calculated via following equations for each item; CVR = (n_E_ – N/2) / (N/2), where n_E_ is the number of specialists who indicate that an item is “essential” and N is the total number of specialists. In order to determine whether to remain or discard specific questions, the CVR values of each item were then compared with the Lawshe table. In the present study, values ≥0.54 were considered reasonable to verify each item [20]. The specialists were also asked to assess the relevance of each questions to measure the CVI. To obtain the CVI value, the expert panel rated the relevance of each questions as 1 (not relevant), 2 (somewhat relevant), 3 (quite relevant), and 4 (very relevant). To this end, the CVI value was calculated using the following formula, CVI = (n/N), where n is the number of specialists who give score of 3 or 4 and N is the total number of experts [21]. Values > 70% were regarded as appropriate to verify each question according to the Lawshe. At the end of this process 4 items were removed yielding a total of 51 items. Then, qualitative method was used for face validity. A group of six 5th-grade girls were asked to examine the questionnaire and indicate whether they could read and understand the questions. As a result, 2 additional items were removed yielding a 49-item provisional version of the questionnaire. As such the total score for the BABAQ range from 16 (lowest) to 132 (highest). We assigned the following criteria to interpret the scores: high (above the third quartile, 104–132); intermediate (between the first and third quartiles, 45–103); and low (less than the first quartile, 16–44).

### Psychometric evaluation

The provisional questionnaire with 49 items [Additional file 1] then was administered to a sample of female students in Tehran Iran. Since previous studies reported higher prevalence and incidence among girls than boys (38·9% vs 35·0%) [3, 9], female students were selected from district 22 where the district represents a population with a variety of socio-economic backgrounds.

### Data analysis

Data was analyzed using the SPSS version 24 software; the level of significance was set at *p* < 0.05. The descriptive statistics was used to present the demographic characteristics of participant and self-reported back and neck pain prevalence during the last week. To assess psychometric properties of the questionnaire the following statistical procedures were applied:

Item analysis: In order to analyze the correlation of items and predefined constructs, item-total correlation analysis was performed. As such the correlation between items and hypothesized constructs was calculated using the Pearson correlation coefficient.

### Structural validity

Confirmatory factor analysis (CFA) was conducted to investigate predefined construct of the BABAQ (see Table 1). The CFA is the best method for evaluating the structural validity of an instrument when there is a theoretical approach to analyze the instrument with specified constructs and for the direct representation of a hypothesized factor model, leading to a measure of model fit [21–24]. Since, in most forms of factor analysis, the assumption is made that the items follow a normal distribution [25] and in this study data were normally distributed, thus for estimation method, maximum likelihood (ML) estimator was applied. To test the goodness-of-fit of the model, the Comparative Fit Index (CFI), Root Mean Squared Error of Approximation (RMSEA), and Standard Root of Mean Square Residual (SRMR) were examined. The data was analyzed using LISREL 8.80 to test for significance of item loadings on each relating factor, and to evaluate overall model fit intended by the SCT framework. The following values were considered acceptable for the model fit: χ2/df < 5, CFI > 0.95, RMSEA < 0.10, SRMR < 0.08 [21]. We also used the Average Variance Extracted (AVE) statistic in order to test the convergent validity of the constructs. The AVE values above 0.50 shows adequate convergent validity.

### Reliability

Internal consistency was estimated using the Cronbach’s alpha coefficient. The value of 0.70 or above was considered satisfactory [26]. The test-retest reliability also was used to examine stability by calculating intraclass correlation coefficient (ICC). A sample of 50 students who did not participate in the main study completed the questionnaire twice within 2 weeks’ interval. The ICC also used to evaluate inter-rater reliability on each group of items for the practical skill domain as rated by two independent and trained raters. Values higher than 0.70 considered excellent agreement [14]. In addition, we estimated the standard error of measurement (SEM). The standard error of measurement (SEM = SD × $\sqrt{1- ICC}$) is an estimate of the amount of error in a test and is directly related to a test’s reliability. The larger the SEM, the lower the test’s reliability. Furthermore, minimal detectable change (MDC) for the BABAQ was calculated. The minimal detectable change (MDC = 1.96 × SEM × $\sqrt{2}$) is the lowest change in the BABAQ score, that ensures the change is not a result of measurement error.

## Results

### Participants

In all, 610 5th-grade girls participated in the study; 50.3% of the participants (*n* = 307) were the only child in family, 74.1% of their father (*n* = 452) and 73.9% of their mother (*n* = 451) had secondary and higher education, respectively; about a quarter of students (*n* = 144) reported back pain during last week. The demographic characteristics of the pupils are shown in Table 2.

**Table 2:** Demographic characteristics of pupils in CFA step (*n* = 610)

	Frequency	Percent
**Father’s job**		
Employed	564	92.4
Unemployed	4	0.7
Retired	28	4.6
Missing	14	2.3
**Mother’s job**		
Employed	123	20.2
Housewife	480	78.7
Missing	7	1.1
**Father’s level of education**		
Illiterate/primary	61	10.0
Secondary	204	33.4
Higher	248	40.7
Missing	97	15.9
**Mother’s level of education**		
Illiterate/primary	79	13.0
Secondary	235	38.5
Higher	216	35.4
Missing	80	13.1
**Birth ranking**		
Being the only child in family	307	50.3
Second child	221	36.2
Ohers	67	11.3
Missing	15	2.5
**Presence of back pain**		
Yes	144	23.6
No	459	75.2
Missing	7	1.1

### Item-total correlation

The correlation between items and predefined constructs are presented in Table 3. As shown the correlation between items and its own predefined construct was satisfactory.

**Table 3:** Item-total correlation matrix for the BABAQ indicating the correlation between items and predefined constructs

	Skills	Knowledge	Self-Efficacy	Beliefs	Behavior
Skills					
checkQ_1_	**0.95**	0.38	0.28	0.13	0.12
checkQ_2_	**0.57**	0.37	0.13	0.17	0.29
checkQ_3_	**0.85**	0.05	0.14	0.28	0.17
checkQ_4_	**0.48**	0.12	0.21	0.05	0.25
checkQ_5_	**0.71**	0.32	0.05	0.18	0.16
checkQ_6_	**0.78**	0.009	0.07	0.19	0.002
checkQ_7_	**0.49**	0.11	0.11	0.21	0.24
checkQ_8_	**0.48**	0.003	0.09	0.07	0.20
checkQ_9_	**0.42**	0.34	0.12	0.33	0.05
checkQ_10_	**0.68**	0.28	0.29	0.35	0.39
checkQ_11_	**0.87**	0.11	0.02	0.22	0.35
checkQ_12_	**0.62**	0.25	0.10	0.12	0.20
checkQ_13_	**0.43**	0.32	0.05	0.29	0.05
checkQ_14_	**0.78**	0.37	0.03	0.37	0.14
checkQ_15_	**0.45**	0.15	0.13	0.25	0.17
checkQ_16_	**0.86**	0.33	0.19	0.32	0.26
checkQ_17_	**0.65**	0.04	0.16	0.06	0.10
checkQ_18_	**0.53**	0.01	0.06	0.05	0.23
checkQ_19_	**0.95**	0.09	0.22	0.01	0.21
checkQ_20_	**0.69**	0.28	0.28	0.16	0.12
checkQ_21_	**0.48**	0.37	0.24	0.12	0.10
checkQ_22_	**0.49**	0.24	0.28	0.28	0.23
checkQ_23_	**0.96**	0.25	0.02	0.15	0.14
Knowledge					
preQ_1_	0.32	**0.74**	0.08	0.19	0.12
preQ_2_	0.35	**0.80**	0.14	0.15	0. 26
preQ_3_	0.19	**0.88**	0.03	0.10	0.27
preQ_4_	0.31	**0.73**	0.11	0.22	0.22
preQ_5_	0.15	**0.87**	0.17	0.26	0.11
preQ_6_	0.09	**0.84**	0.10	0.07	0.15
preQ_7_	0.33	**0.77**	0.01	0.08	0.23
preQ_8_	0.14	**0.93**	0.07	0.13	0.26
preQ_9_	0.12	**0.84**	0.03	0.16	0.16
preQ_10_	0.09	**0.77**	0.32	0.32	0.01
Self-Efficacy					
preQ_11_	0.21	0.21	**0.81**	0.09	0.15
preQ_12_	0.19	0.15	**0.79**	0.03	0.06
preQ_13_	0.39	0.01	**0.92**	0.11	0.19
preQ_14_	0.26	0.14	**0.87**	0.12	0.37
Beliefs					
preQ_15_	0.29	0.11	0.12	**0.86**	0.19
preQ_16_	0.26	0.17	0.09	**0.75**	0.13
preQ_17_	0.03	0.28	0.11	**0.86**	0.03
preQ_18_	0.01	0.14	0.19	**0.76**	0.27
preQ_19_	0.07	0.12	0.10	**0.76**	0.20
preQ_20_	0.09	0.23	0.04	**0.83**	0.16
Behavior					
preQ_21_	0.13	0.07	0.11	0.07	**0.80**
preQ_22_	0.12	0.04	0.24	0.39	**0.79**
preQ_23_	0.06	0.01	0.04	0.19	**0.89**
preQ_24_	0.10	0.15	0.34	0.04	**0.93**
preQ_25_	0.17	0.13	0.23	0.19	**0.86**
preQ_26_	0.16	0.12	0.26	0.18	**0.73**

### Structural validity

**Fig. 1:**
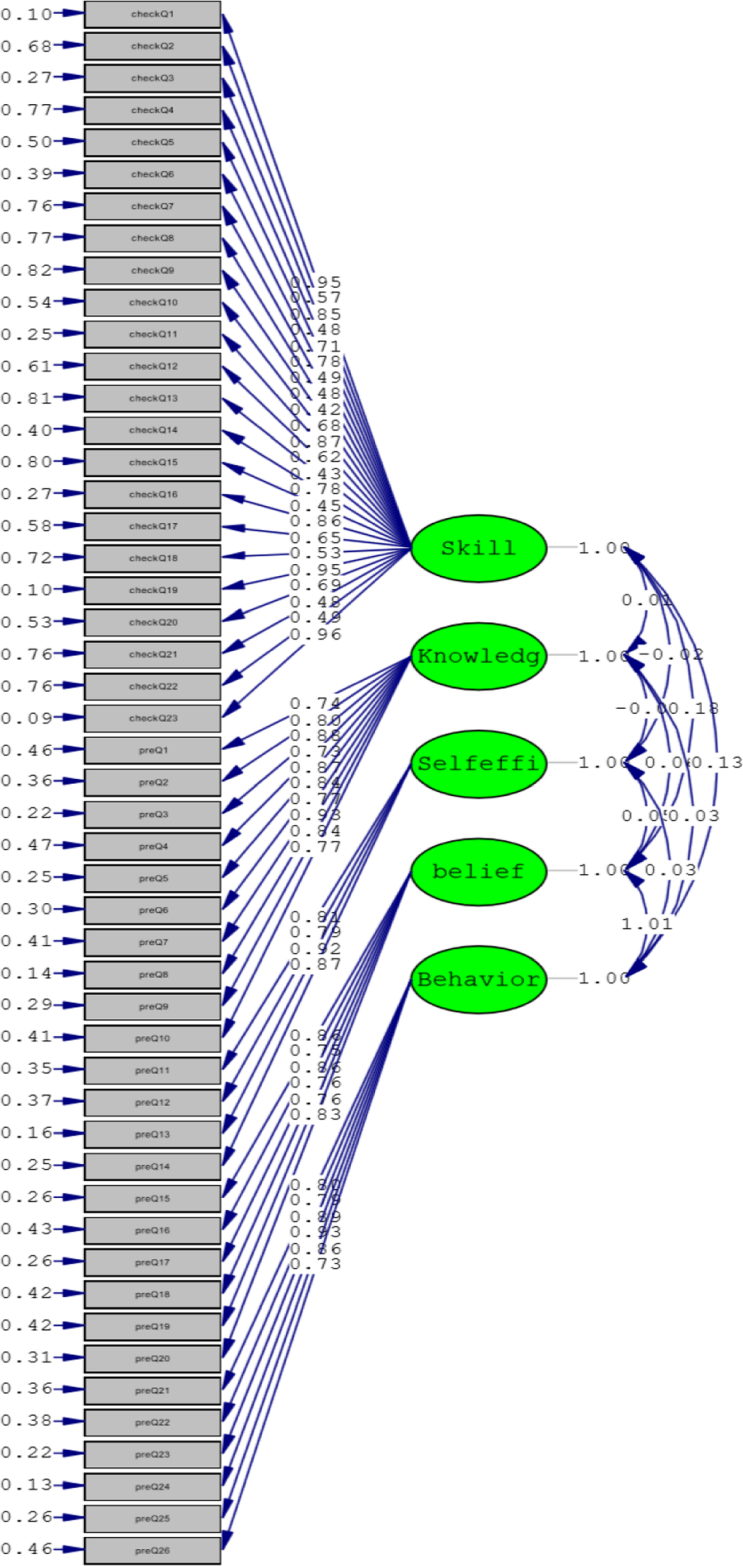
The results ontained form confirmatory factor analysis for the BABAQ

The results obtained from confirmatory factor analysis for the BABAQ were as follows: Chi-Square = 3921.78, df = 1117 (χ2/df = 3.51, *P* = 0.53), CFI = 0.97, RMSEA = 0.091 (*P* < 0.001), and SRMR = 0.078. Figure 1. shows the factor weighting value results in the standard estimation mode.

### Reliability

The Cronbach’s alpha coefficients for all subscales were high ranging from 0.93 to 0.97. The intraclass correlation coefficient of the four self-reported subscales of the BABAQ ranged from 0.76 to 0.83. Table 4 represents the Cronbach’s alpha coefficients, ICC values, SEM, and MDC for the questionnaire.

**Table 4:** Descriptive statistics, Cronbach’s alpha coefficient, ICC, SEM, and MDC for the BABAQ

Subscale [number of items]	Possible score range	Mean (SD)	Cronbach’s alpha	ICC	SEM	MDC
Skills [23]	0–46	13.09 (14.56)	0.97	0.78	4.96	13.74
Knowledge [10]	0–10	4.56 (1.44)	0.96	0.76	0.72	1.99
Self-Efficacy [4]	4–16	11.74 (3.05)	0.93	0.83	1.42	3.94
Expectation beliefs [6]	6–30	20.43 (4.84)	0.94	0.80	2.49	6.90
Behavior [6]	6–30	21.89 (5.22)	0.95	0.83	2.33	6.46
Total [49]	16–132	72.28 (16.96)	0.93	0.84	7.08	19.62

*ICC* Intraclass correlation coefficient, *SEM* Standard error of measurement, *MDC* Minimal detectable change

### Convergent validity

The calculated Average Variance Extracted (AVE) values for skills, knowledge, self-efficacy, beliefs, and behavior were 0.54, 0.73, 0.79, 0.49, and 0.86 respectively indicating adequate convergent validity, although expectation beliefs had AVE value close to 0.50. In addition, we estimated values for the skills subscale inter-rater agreement (Table 5).

**Table 5:** Intraclass correlation coefficient for Skills (checklist) inter-rater agreement (*n* = 50)

	ICC (%)
***Sitting at a table***	
checkQ1. Straight, not slouched	0.95
checkQ2. Feet on the floor	0.88
checkQ3. No twisting	0.94
***Pick up the crate***	
checkQ4. Wide base of support	0.92
checkQ5. Load close	0.85
checkQ6. Bend knees	0.75
checkQ7. Back straight	0.88
checkQ8. No twisting	0.89
***Carry the crate***	
checkQ9. Back straight (not swayed)	0.93
checkQ10. Load close/elbows bent	0.89
***Set the crate down on the table***	
checkQ11. Bend knees	0.74
checkQ12. Load close	0.84
***Pick up a pencil***	
checkQ13. Wide base of support	0.85
checkQ14. Bend knees	0.73
checkQ15. Back straight	0.81
***Move the crate***	
checkQ16. Back straight	0.90
checkQ17. Load close & in front	0.88
checkQ18. Step/pivot not twist	0.74
***Backpack***	
checkQ19. Load correctly (order)	0.95
checkQ20. Handling the bag (bend knees)	0.89
checkQ21. Handling the bag (wide base)	0.84
checkQ22. Handling the bag (back straight)	0/90
checkQ23. Carrying the bag 2 straps	0.78

## Discussion

This study is a modest contribution to ongoing discussions on development and psychometric testing of the Back-Care Behavior Assessment Questionnaire (BABAQ) among 5th-grade girls in some Iranian elementary schools. Particular attention is thus paid to measure validity and reliability of the BABAQ sub-scales. For a few reasons, this study has a novel approach and is important. First, the originality of this study lies in the fact that it is a theory-based instrument in evaluating healthy spine-related behaviors in pupils. It is also significant because the BABAQ can provide the opportunity to assess behaviors and their determinants according to the Social Cognitive Theory (SCT). As such, the instrument developed might help create a theory-based intervention in order to change unsafe behaviors among pupils. Secondly, the psychometric properties of the BABAQ are evaluated while four groups including the research team (academics), the 5th-grade girls, their teachers, and health specialists are involved. Thirdly, to the best of authors’ knowledge, this is the first attempt reporting on construct validity of an instrument for back pain prevention, employed for evaluating education programs.

Content validity verification in this study indicated that three items associated with knowledge section including ‘Who is sitting the best way’, ‘If you have to move equipment in the gym, you should ...’, and ‘Which posture is the best?’ had no acceptable values. As well, one item related to behavior section, i.e., ‘No twisting while moving heavy objects’ had the same conditions. Accordingly, all the mentioned items removed from the final version. The panelists also believed that these items were irrelevant. However, these results are in good agreement with Dolphens et al., using almost similar items in their questionnaires [9].

The further contribution of this study is recruiting construct validity and CFA to test multiple variables, while there was a theoretical framework [20]. Moreover, various indicators such as the Chi-square (χ2)/degree of freedom (df) ratio, the comparative fit index (CFI), the standardized root mean square residual (SRMR), and the root mean square error of approximation (RMSEA) RMSEA verified the fitness of the models. In addition, the findings demonstrated that each of the five sub-scales in the BABAQ had appropriate fit within the SCT framework.

Empirical results from the Cronbach’s alpha, test-retest, and inter-rater reliability also confirmed that the BABAQ showed acceptable internal consistency (ranged from 0.93 to 0.97) within the five sub-scales, providing reliable results over repeated administrations (ranged from 0.76 to 0.83), and producing significant inter-rater agreement (ranged from 0.73 to 0.95) at the 5th-grade level. Likewise, the higher values of the BABAQ scores were associated with greater standard deviations (SDs) (expected knowledge), accounting for the remarkably higher standard error of measurement (SEM) scores for each sub-scale. The higher scores for the BABAQ could be due to the small sample size in this study. In previous studies, the reliability of the questionnaires had been assessed only from the aspect of test-retest stability and internal consistency. For example, Cardon et al. had evaluated different instruments, based on previous literature, indicating reliability ranged from 0.42 to 0.82 [13]. Cronbach’s alpha coefficient of the expectation beliefs was also 0.70 and other intended sections were not applicable in the present study. In order to verify face and content validity, 150 children, 20 parents, and 10 teachers had completed the questionnaire to identify unclear items, which had been then modified. Moreover, they had not used panelists. Inter-rater reliability results in the present study are accordingly in relative agreement with the findings reported by Cardon et al., obtaining the intra-class correlation coefficient (ICC) to determine inter-rater agreement on the sum scores of the practical test items, ranged from 0.785 to 0.980 [14]. Other results were also better than previous studies.

It is argued that the BABAQ is suitable for a wide variety of potential applications to measure back-care behaviors and their main determinants among the 5th-grade girls. One unique feature of the BABAQ is the reliability and validity of its sub-scales, which contain back-care skills and knowledge, self-efficacy towards proper back-care behaviors, expectation beliefs, and healthy spine-related behaviors. These sub-scales may be measured, evaluated, and modified by potential change strategies, thereby providing back pain prevention and ultimately back health promotion.

### Limitations

In this study, there are limitations that must be noted. First, data were only collected from the 5th-grade girls’ population attending public elementary school in capital Tehran’s region 22; and other independent elementary schools, grades, as well as male pupils didn’t enroll to study; therefore, the generalizability of outcomes to the overall population may be limited. In addition, due to decrease recall bias, back pain report was limited within the last week. Subscales of the BABAQ were limited to main psychological determinants of behavior in SCT and the other constructs (environmental determinants of behavior), in other to decrease the questions’ burden on participants, didn’t use. In skills items construct validity verification phase, sample was limited to fewer population because difficulty of assessing. However, future studies should test CFA with an adequate number of participants. Despite these limitations that have been explained, the BABAQ is a valid and reliable instrument to measure healthy spine-related behavior in girls as young as 11 years of age.

## Conclusion

The Back-care Behavior Assessment Questionnaire (BABAQ) demonstrated to be a valid instrument to measure healthy spine-related behavior including behavioral capability (skills and knowledge), self-efficacy, expectation beliefs and performance spine. Future attempts should focus on to assess whether the BABAQ is applicable in diverse pupils’ populations.

## Supplementary information

**Additional file 1.** Back-care Behavior Assessment Questionnaire.

## Data Availability

The datasets used and analyzed during the current study are available from the corresponding author on reasonable request.

## Abbreviations

AVE: Average Variance Extracted
BABAQ: Back-care Behavior Assessment Questionnaire
CFA: Confirmatory factor analysis
CFI: Comparative fit index
CVI: Content validity index
CVR: Content validity ratio
ICC: Intraclass correlation coefficient
MDC: Minimal detectable change
MSDs: Musculoskeletal disorders
ML: Maximum likelihood
RMSEA: Root mean squared error of approximation
SCT: Social cognitive theory
SE: Self efficacy
SEM: The standard error of measurement
SRMR: Standard Root of Mean Square Residual
