# Supplementary material for: The Back-care Behavior Assessment Questionnaire (BABAQ) for schoolchildren: development and psychometric evaluation
Source: BMC Public Health. 2020 Aug 26;20:1283. doi: 10.1186/s12889-020-09318-9 (PMC7448470; doi:10.1186/s12889-020-09318-9)
Supplement: Supplementary file 1 — Additional file 1. Back-care Behavior Assessment Questionnaire. [file 12889_2020_9318_MOESM1_ESM.docx]

**Back-care Behavior Assessment Questionnaire (BABAQ)**

**Dear Student,**

We are concern about your health. Specifically, we are concern about you low back pain. We would be very grateful if you could respond to the following questions as appropriate. We hope this information could help us to plan interventions that might improve student’s health a quality of life.

Please consider:

- If you do not know an answer, please write your best guess.
- There is no time limit, so please take your time.

| School: —— | Class number: —— | Pupil code: —— |
| --- | --- | --- |

| **Knowledge items (score range: 0-10, pass/fail scoring procedure)** | | | | | | | | |  |
| --- | --- | --- | --- | --- | --- | --- | --- | --- | --- |
| preQ1. When lifting a heavy box off the floor: | | | | | | | | |  |
| a) You should keep your feet as far apart as possible. | | | | | | | | |  |
| b) You should do most of the work with your back. | | | | | | | | |  |
| c) You should bend your knees. | | | | | | | | |  |
| d) You should keep the box on one side of your body. | | | | | | | | |  |
| preQ2. The best way to carry your groceries is: | | | | | | | | |  |
| 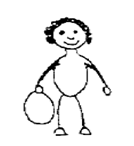  In one big bag | | | 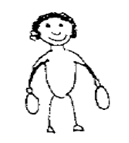  In two bags | |  | | | |  |
| preQ3. Which is the best way to carry your book bag? | | | | | | | | |  |
| 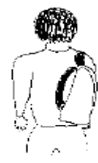  Over one  shoulder | | 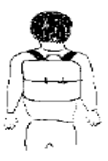  Over two  shoulders | | 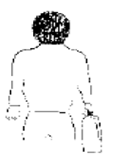  In one  hand | | | |  |  |
| preQ4. When carrying a box, it is best to carry it: | | | | | | | | |  |
| 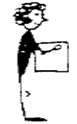  As close as possible  to your body | | 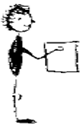  A little further from your body | | 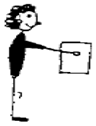  As far as possible from your body | | |  | |  |
| preQ5. If you pick up books from a pile on the floor and put them on a table: | | | | | | | | |  |
| a) You should not move your feet and just turn your body. | | | | | | | | |  |
| b) You should move your feet each time. | | | | | | | | |  |
| c) You should move your feet just a little and then turn your body. | | | | | | | | |  |
| preQ6. The hardest position for your back is: | | | | | | | | |  |
| a) Laying down on your side | | | | | | | | |  |
| b) Sitting | | | | | | | | |  |
| c) Standing up | | | | | | | | |  |
| d) Lying on your back | | | | | | | | |  |
| preQ7. A spine: | | | | | | | | |  |
| a) Has no curves. | | | | | | | | |  |
| b) Has 2 curves. | | | | | | | | |  |
| c) Has 3 curves. | | | | | | | | |  |
| d) Has 4 curves. | | | | | | | | |  |
| preQ8. During your playtime, it’s best for your back if you: | | | | | | | | |  |
| a) Sit down | | | | | | | | |  |
| b) Move a lot | | | | | | | | |  |
| c) Stand still | | | | | | | | |  |
| preQ9. Which book bag is loaded in the best way? | | | | | | | | |  |
| 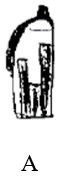 | 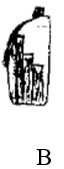 | | 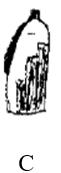 | 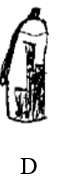 | |  | | | |
| preQ10. How much should the maximum weight of your book bag be? | | | | | | | | |  |
| a) Your own weight | | | | | | | | |  |
| b) Your bodyweight, divided by 2 | | | | | | | | |  |
| c) Your bodyweight, divided by 5 | | | | | | | | |  |
| d) Your bodyweight, divided by 10 | | | | | | | | |  |

**References:**

1. Sheldon MR. Lifting Instruction to Children in an Elementary School. J Orthop Sport Phys Ther. 1994;19(2):105–10.

2. Cardon G, De Clercq D, De Bourdeaudhuij I. Effects of back care education in elementary schoolchildren. Acta Paediatr. 2000;89(6):1010–7.

3. Cardon G, Bourdeaudhuij L., Clercq D. Knowledge and Perceptions About Back Education Among Elementary School Students, Teachers, and Parents in Belgium. J School Health. 2002;72(3):100–6.

4. Heiser, Leigh S, Belcher D, Anshel M, Fuller D. Effects of a back pain prevention education program on knowledge of proper back care among fifth grade elementary students. ProQuest Dissertations and Theses [Internet]. 2014. Retrieved from <http://search.proquest.com/docview/>1545616059

| **Self-efficacy items (score range: 4-16) *** | | | | |
| --- | --- | --- | --- | --- |
|  | Difficult | Almost difficult | Almost easy | Easy |
| preQ11. How do you perceive participating in physical activity and sports each day? |  |  |  |  |
| preQ12. How do you perceive attaining a natural curvature of the spine? |  |  |  |  |
| preQ13. How do you perceive checking your book bag weight? |  |  |  |  |
| preQ14. How do you perceive paying attention to ergonomically postures? |  |  |  |  |

* Higher scores denoted higher self-efficacy.

**References:**

1. Cardon G, Bourdeaudhuij L., Clercq D. Knowledge and Perceptions About Back Education Among Elementary School Students, Teachers, and Parents in Belgium. J School Health. 2002;72(3):100–6.

2. Dolphens MBC, Danneels LDDC, Ilse De Bourdeaudhuij Greet Cardon. Long-term effectiveness of a back education programme in elementary schoolchildren: an 8-year follow-up study. Eur Spine. 2011; 20:2134–42.

| **Expectation beliefs items (score range: 6-30) *** | | | | | |
| --- | --- | --- | --- | --- | --- |
|  | Strongly disagree | Disagree | Neutral | Agree | Strongly agree |
| preQ15. When having a backache, swimming is dangerous. |  |  |  |  |  |
| preQ16. When having a backache, sitting is dangerous. |  |  |  |  |  |
| preQ17. When having a backache, running is dangerous. |  |  |  |  |  |
| preQ18. When having a backache, participating in physical activity is dangerous. |  |  |  |  |  |
| preQ19. When having a backache, cycling is dangerous. |  |  |  |  |  |
| preQ20. When having a backache, lifting heavy objects is dangerous. |  |  |  |  |  |

* Higher scores denoted higher beliefs.

**References:**

1. Cardon G, Bourdeaudhuij L., Clercq D. Knowledge and Perceptions About Back Education Among Elementary School Students, Teachers, and Parents in Belgium. J School Health. 2002;72(3):100–6.

2. Dolphens MBC, Danneels LDDC, Ilse De Bourdeaudhuij Greet Cardon. Long-term effectiveness of a back education programme in elementary schoolchildren: an 8-year follow-up study. Eur Spine. 2011; 20:2134–42.

| **Back care** **behavior items** **(score range: 6-30) *** | | | | | |
| --- | --- | --- | --- | --- | --- |
|  | Never | Hardly ever | Sometimes | Almost ever | Ever |
| preQ21. Do you check the weight of your book bag? |  |  |  |  |  |
| preQ22. Do you carry the bag with 2 straps? |  |  |  |  |  |
| preQ23. Do you do exercise every day? |  |  |  |  |  |
| preQ24. Do you have knee position when putting on shoes? |  |  |  |  |  |
| preQ25. Do you have knee position when lifting? |  |  |  |  |  |
| preQ26. Do you carry an object with the load close to the body? |  |  |  |  |  |

* Higher scores denoted higher behavior.

**References:**

1. Cardon G, Bourdeaudhuij L., Clercq D. Knowledge and Perceptions About Back Education Among Elementary School Students, Teachers, and Parents in Belgium. J School Health. 2002;72(3):100–6.

2. Dolphens MBC, Danneels LDDC, Ilse De Bourdeaudhuij Greet Cardon. Long-term effectiveness of a back education programme in elementary schoolchildren: an 8-year follow-up study. Eur Spine. 2011; 20:2134–42.

**Score sheet for the skills assessment (score range: 6-46)**

**References:**

1. Cardon G, De Clercq D, De Bourdeaudhuij I. Effects of back care education in elementary schoolchildren. Acta Paediatr. 2000;89(6):1010–7.

2. Heiser, Leigh S, Belcher D, Anshel M, Fuller D. Effects of a back pain prevention education program on knowledge of proper back care among fifth grade elementary students. ProQuest Dissertations and Theses [Internet]. 2014. Retrieved from <http://search.proquest.com/docview/>1545616059
